# The challenges of balancing clinical and research responsibilities

**DOI:** 10.1590/0100-3984.2023.0119

**Published:** 2024-04-15

**Authors:** Nikolaos-Achilleas Arkoudis, Evgenia Efthymiou, Ornella Moschovaki-Zeiger, Christos Koutserimpas

**Affiliations:** 1 Research Unit of Radiology and Medical Imaging, 2nd Department of Radiology, Medical School, National and Kapodistrian University of Athens, Athens, Greece; 2 2nd Department of Radiology, University General Hospital of Athens “Attikon”, Medical School, National and Kapodistrian University of Athens, Athens, Greece; 3 Department of Orthopaedics and Traumatology, “251” Hellenic Air Force General Hospital of Athens, Athens, Greece

Research plays an indispensable role in the field of medicine, driving the advancement of health care and facilitating the development of novel and effective approaches for diagnosing, monitoring, and treating diseases^(1)^. By sharing their research outcomes, medical professionals contribute to the collective knowledge base, promoting a continuous cycle of learning, refinement, and advancement within the medical field. In addition, published research often serves as a reference for future studies, building upon existing information and driving further scientific exploration^(1)^. Furthermore, publishing research manuscripts brings recognition and credibility to medical professionals; it allows them to showcase their expertise and contributions, enhancing their professional reputation and career growth opportunities^(2)^. Consequently, the publication of research manuscripts has progressively become an essential requirement intertwined with the careers of medical professionals. However, due to immense competition, physicians may have to continuously increase the number of publications they produce in order to succeed and advance in their field, a phenomenon otherwise referred to as the “publish or perish” mentality^(3)^. Beyond the production of the actual research, the publication process itself can often be time-consuming, involving multiple rounds of peer review and revisions by physicians who already have limited spare time. It is not uncommon for them to have to work diligently during weekends or outside regular working hours to fulfill their responsibilities, such as completing research projects, conducting peer reviews, and meeting critical deadlines. Juggling research and clinical work under these conditions can become overwhelming and may lead to unorthodox time management strategies and ineffective prioritization. The combination of the above can significantly increase the risk of burnout syndrome, which has been observed in up to one-third of health care workers at any given point in time^(4)^. This concerning fact underscores the potential for serious repercussions, including a diminished standard of clinical care. That raises an important question: Is the need for rigorous and intensive publication overshadowing the other tasks to which physicians devote their time, such as their primary clinical responsibilities, thereby leading to a decline in the quality of services provided to patients?

This issue is particularly relevant for health care professionals who are actively engaged in patient care while also conducting research. Those who must focus on clinical care daily and work long hours may not have the time or energy to conduct or be part of research. Some may therefore choose not to prioritize that task, whereas others, either because of personal aspirations or because of a desire not to sell themselves short on future career opportunities, may choose to be occupied with both. That could lead physicians to shift part of their focus or working hours from clinical care to academic writing and publishing. This poses a significant threat, particularly in hospitals and institutions that are already struggling with understaffing issues. In addition, in some countries, research and pursuing a doctoral degree may be compensated inadequately^(5)^ or not at all. In such contexts, health care professionals may have no option but to pursue their research goals parallel to their regular, compensated clinical duties, which can further exacerbate the challenges of both.

Although publishing research is important, it should not be at the expense of providing adequate patient care. Is it possible to address this matter in a way that benefits both patient care and the advancement of medical knowledge?

Up to a point, these issues could be addressed by physicians on a personal level. Successful time management and prioritization are crucial skills that need to be developed in order to strike a balance between these responsibilities. In addition, fostering collaborations among peers, engaging in collaborative research efforts, and delegating tasks can help alleviate the time constraints physicians face. Distributing the workload and sharing responsibilities may create more time for dedicated patient care. Furthermore, participating in research directly aimed at patient care, such as clinical trials or quality improvement standards, can be a valuable approach because it will allow research to be conducted within the health care setting itself, generating publishable data while contributing to patient care. However, to address this matter respectfully, broad reforms may also be called for. Health care facilities and organizations should provide dedicated research time, incorporated into regular working hours, thus recognizing the importance of research as an integral part of the role played by health care professionals. Allocating specific time for research within their regular schedules would preclude the need for those professionals to seek compromises between their clinical duties and their research or to work outside regular hours. Moreover, institutions should provide support and resources to clinicians engaged in research, including access to research facilities and assistance with data analysis and manuscript preparation. These seem like essential steps toward addressing the challenges of efficiently juggling clinical and research responsibilities. Lastly, in addition to traditional academic publishing, it is crucial that alternative forms of scholarly activity are acknowledged. For example, teaching, educating, and mentoring younger physicians or peers, which might be currently overlooked due to the difficulty in measuring the impact of those activities in comparison with the quantifiable nature of publications^(3)^, should in due course receive the recognition they deserve.

In conclusion, the tension between the demands of clinical practice and the mandate to publish appears to be more significant and pressing than ever before. The perspectives raised above have long constituted an issue of concern yet remain largely unaddressed. Neglecting to confront this issue will merely delay its resolution, and we should instead be seeking a means of resolving it effectively and permanently.
